# HMGB-1 in Psoriasis

**DOI:** 10.3390/biom12010060

**Published:** 2021-12-31

**Authors:** Marco Casciaro, Eleonora Di Salvo, Sebastiano Gangemi

**Affiliations:** 1 School and Unit of Allergy and Clinical Immunology, Department of Clinical and Experimental Medicine, University of Messina, 98125 Messina, Italy; gangemis@unime.it; 2 Department of Veterinary Sciences, University of Messina, 98168 Messina, Italy; edisalvo@unime.it

**Keywords:** HMGB-1, psoriasis, alarmin, skin, inflammation, immune system, monoclonal, biologic, therapy

## Abstract

Psoriasis is a multifactorial pathology linked to systemic inflammation. Enhanced keratinocytes proliferation and a minor maturation state of the cells are typical features. Perivascular T cells, dendritic cells, macrophages, and neutrophilic granulocytes are part of the scenario completed by apoptosis dysregulation. Several proinflammatory mediators, alarmins and growth factors are increased too, both in the skin and the patients’ blood. HMGB1 is important as an alarmin in several inflammatory conditions. Released after cellular damage, HMGB1 acts as a danger signal. Several studies have considered its role in psoriasis pathogenesis. We evaluated its level in psoriasis and the potential of the alarmin blockade through standard therapies, biological treatments and using monoclonal antibodies. PV patients were shown to have significantly increased levels of HMGB1 both in lesional skin and in serum, which were linked, in some cases, to other pro-inflammatory markers and alarmins. In most cases these parameters were correlated with PASI score. Data demonstrated that blocking HMGB1 is effective in ameliorating psoriasis. Focusing on this approach could be valuable in terms of a therapeutic option for counteracting immune-related diseases in a way unthinkable until few years ago.

## 1. Introduction

Psoriasis is an autoimmune skin disease characterized by a dysregulation of the immune system with multifactorial pathogenesis. The etiology of the disease is still controversial but biochemical, genetic, and environmental factors are fundamental for disease initiation and worsening [1]. This multifactorial pathology is linked to systemic inflammation. Enhanced keratinocytes proliferation and a minor maturation state of these cells is a typical feature [2]. Immune cells infiltration, perivascular T cells, dendritic cells, macrophages, and neutrophilic granulocytes are part of the scenario, which is completed by apoptotic dysregulation. Several proinflammatory mediators, alarmins and growth factors are also increased too, both in skin and in the patients’ blood [3].

### Alarmins and Skin

Interleukin 33 (IL-33) is a molecule discovered in the last decade, which is part of the IL-1 family. It has multiple functions, such as the stimulation of IL-4, IL-5 and IL-13, which are T helper 2 (Th2) cytokines. IL-33 links to the interleukin 1 receptor-like 1 protein (also IL-1RL1 or ST2). Several cells express IL-33. Among these there are fibroblasts, epithelial cells, endothelial cells and activated macrophages. IL-33 has also been recently reported to act also as a danger signal (alarmin) [4]. IL-33 is usually intimately linked to chromatin and is most often represented in cells with a barrier function, (i.e., endothelial and epithelial cells) [5]. A biologically active form of IL-33 is released when these cells are destroyed after damage. For this reason, IL-33 is like other biologically active mediators such as S100 proteins, IL-1β and the high-mobility group box 1 (HMGB1) [6].

S100 proteins have intracellular and extracellular functions. Although intracellular capabilities of the molecule have been clearly shown, extracellular functions are still being investigated. S100 proteins are usually released after cell damage. S100A4 and A6 can promote apoptosis, S100B, S100A8/A9, S100A11 and S100A12 inflammation, S100A8 and A9 chemotaxis, and S100P and S100A7 cell proliferation [7]. One molecule important as an alarmin in several inflammatory conditions is HMGB1. It is also called amphoterin, and is a highly conservative damage-associated molecular pattern molecule. It has a dual function. In the nucleus, it regulates nuclear biochemical transactions. Once released after cellular damage, HMGB1 acts as a danger signal resulting from local wounding [8]. HMGB1 has a key role in the human metabolome [9]. As mentioned above there is ever-increasing evidence of its involvement in immunopathology in diverse organs [10,11]. Several studies have considered its role in the pathogenesis of psoriasis, and for this reason we decided to focus on the role of HMGB1 in this skin disease. Particularly, we decided to evaluate the potential of the alarmin blockade using standard therapies, biological treatments and specific monoclonal antibodies.

## 2. Materials and Methods

A review was performed by browsing the PubMed and Scholar databases, searching from inception until 30 October 2021. The search included terms related to psoriasis and high mobility group box-1 (HMGB-1).

We read the abstracts of articles with titles suggesting an association between psoriasis and alarmins. The entire article was read only if the abstract indicated that the article potentially met the inclusion criteria (research article, trials, English-written). Finally, we reviewed and searched the references of these articles to identify further studies which could be included. We included in this perspective a total of 10 research articles.

## 3. Results

Literature data linking psoriasis to HMGB1 alarmin starts in 2013 with Chen et al. Since that first association, other research followed analyzing serum, and lesional skin levels of the alarmin. Researchers observed HMGB1 effects on keratinocytes by using recombinant proteins (Table 1) and some focused on the effects of the HMGB1 blockade (Table 2).

### 3.1. HMGB-1

Chen et al. reported for the first time that circulating levels of HMGB1 in PV patients were higher than in healthy subjects. Blood HMGB1 was correlated with PV severity according to PASI scores. High cytoplasmic HMGB1 levels in PV lesional skin were also reported. Immunohistochemistry and immunofluorescence demonstrated the presence of HMGB1 only in the nucleus of normal skin. HMGB1 was expressed in the nucleus of healthy subjects but not in the cytoplasm [20].

Imiquimod (IMQ) induced psoriasis-like inflammation was exposed to recombinant HMGB1 and to phosphate-buffered saline (PBS) i.d. Lesional skin from IMQ-treated mice presented high cytoplasmic levels of HMGB1 and the administration of HMGB1 into the lesional skin further worsened the psoriasis-like disease. As a result, HMGB1 augmented the infiltration of CD3+ T cells, myeloperoxidase + neutrophils and CD11c+ dendritic cells, increased the number of cd T cells, and upregulated the mRNA expression of interleukin (IL)-6, tumor necrosis factor (TNF)-a, interferon (IFN)-c and IL-17 [12].

Watanabe et al. studied the expression of HMGB-1 in generalized pustular psoriasis (GPP). An increase in HMGB-1 expression was significantly higher in GPP patients [13]. Borsky et al. found significantly increased levels of HMGB1 but focused on PV. They found a positive relationship between HMGB1 and S100A7 [5].

Wang et al. injected wild-type (WT) mice intradermally with recombinant HMGB1 (rHMGB1). The mice tested underwent epidermal thickening and cell infiltration. rHMGB1 triggered inflammatory mediators associated with PV, such as: Cxcl1, Cxcl2, and Ccl20, S100a7, S100a8, S100a9, and Defb2 (defensin beta 2), IL1b and TNF, IL23A-IL17A axis cytokines IL23a, IL17a, and IL22, transcription factors (Rorc/Rorγt) and the neutrophil membrane marker Ly6g [21].

Kamel et al. evaluated serum HMGB1 in PV and correlated it with disease severity. Moreover, they compared the levels in PV patients with and without metabolic syndrome. They realized that HMGB1 was higher in patients than in controls, and was correlated with disease activity. Metabolic syndrome was associated with high levels of the alarmin [15]. 

Wang et al. observed a positive HMGB1 representation in the nucleus of lesional skin; whereas they reported a weak positive presence in the cytoplasm of the squamous epithelium. Extracellular HMGB1 was only rarely present in the epithelium intercellular spaces. They also found that the nucleus and cytoplasm of associated inflammatory cells and vascular endothelial cells were mildly positive to HMGB1. However, HMGB1 levels in epithelial intercellular spaces in psoriasis were increased more than in controls [16].

Zhang et al. investigated if HMGB1 had proinflammatory effects on keratinocytes and how could it contribute to PV development. Human keratinocytes were tested with recombinant human HMGB1. The authors evaluated the variation of inflammatory factors and intermediary signaling pathways. Several inflammatory factors (a total of 11) were triggered and upregulated by HMGB1 in keratinocytes. Interleukin (IL)-18 showed the most significant elevation. The activation of the nuclear factor-B pathway and inflammasomes were connected to the production and release of IL-18 induced by HMGB1. Moreover, the imiquimod-induced psoriasis mice model was used to check the role of HMGB1 in PV equilibrium in vivo. HMGB1, and subsequently IL-18, determined the development of psoriasiform dermatitis in the imiquimod-treated mouse model [17].

Bergmann et al. demonstrated that extracellular serum HMGB1 level affected by severe PV was higher than in healthy controls. They showed that the increase was linked to disease progression [18].

Strohbuecker et al. observed high positive staining for HMGB1 in the cutis of psoriatic lesions compared to the healthy skin of PV patients. Moreover, both the major histocompatibility complex class III-encoded DNA and HMGB1 RAGE, endorsed by HMGB1, were abundantly represented on psoriatic CD8+ T cells and CD4+ Treg. High expression of HMGB1 and RAGE were reported on severe PV plaques [19].

### 3.2. Alarmin Blockade

Chen et al., using an anti-HMGB1 monoclonal antibody and the HMGB1 inhibitor glycyrrhizin, reported that HMGB1 blockade resulted in a diminished number of cd T cells. Another result was the suppression of IL-6, TNF-a, IFN-c and IL-17 mRNA. This scenario was reflected by a mild clinical and histological progression in the IMQ-treated skin [12]. Watanabe et al. reported that serum HMGB-1 levels were significantly decreased after systemic treatment with TNF-a inhibitors or with granulocytapheresis. The authors reported a high positive correlation between serum HMGB-1 levels and the Japanese severity score for GPP [13]. Wang et al. generated Hmgb1-KD animals by the subcutaneous administration of a lentivirus expressing a small hairpin RNA (shRNA) versus Hmgb1 (Hmgb1 shRNA). IMQ induced psoriasis-like inflammation was significantly reduced in KD mice compared to WT mice. Clinical parameters, such as PASI, were sustained by mice epidermal thickening and inflammation, and downregulation of psoriasis-related genes. Blocking extracellular HMGB1 led to comparable results. It was speculated that T-helper 17 immune response in the IMQ model could be blocked by both HMGB1 and IL-18 inhibition by the administration of neutralizing antibodies. This treatment resulted in a much thinner epidermis than in controls [14]. According to Bergamnn et al., patients who underwent methotrexate administration showed the lowest circulating levels of HMGB1. Patients treated with TNFα-inhibitors or fumaric acid esters also had reduced levels of circulating HMGB1. Among the treatments evaluated, only IL-12/IL-23 inhibitors did not influence blood HMGB1 [18]. Another experiment focused on the injection of HMGB1-neutralizing antibodies in psoriatic mice peritoneum. The authors reported a diminished number of infiltrating CD3+ T cells and CD4+ RORγt+Th17 cells in skin lesions and reduced circulating levels of IL-17. According to their results, HMGB1 sustained the Th17 immune response in PV via caspase 1 cleavage and, in turn, the release of IL-18 from keratinocytes [17].

## 4. Discussion

The alarmin, HMGB1, as a nuclear binding molecule is intimately connected with chromatin. It is secreted from several immune cells or accidentally released from cells after permanent damage, acting as a proinflammatory cytokine. It exerts its inflammatory activity by interacting with multiple receptors such as the receptor for advanced glycation end products (RAGE), the Toll-like receptor (TLR) 2 and TLR 4. The alarmin, in turn, stimulates the release of other proinflammatory mediators such as TNFα and IL-1β, resulting in cell damage which, releases more HMGB1 in an autocrine loop. As explained below, the redox status of HMGB1 has main role in immunological activity during damage-associated secretion. However, a unique trigger of PV is undetectable. HMGB1, which is secreted by keratinocytes and immune cells after certain damage or situations, plays a main role in PV immunopathogenesis by influencing phagocytosis and the differentiation of regulatory T cells into IL-17-producing cells [18].

**Figure 1:**
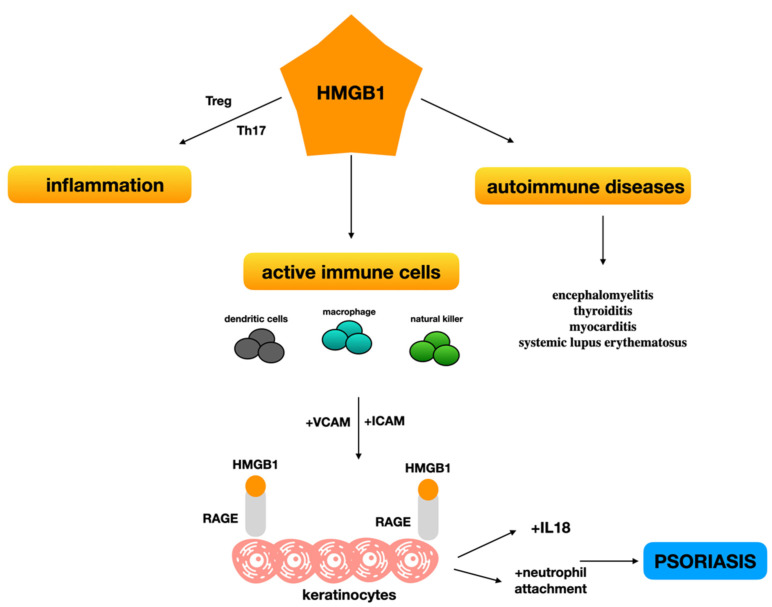
HMGB-1 involvement in the inflammatory process endorsing psoriasis plaque formation.

HMGB1 could function as a proinflammatory cytokine; as a result, it contributes to the development of psoriasis-like inflammation [12]. The presence of this alarmin influences the balance of chronic inflammation in psoriasis with a particular involvement of Treg and Th17cells [20]. HMGB1 plays its role in several autoimmune diseases such as encephalomyelitis, thyroiditis, myocarditis, systemic lupus erythematosus and many others [22,23,24,25]. The alarmin is involved in this specific case in PV because of its release by keratinocytes and diverse immune cells, such as activated macrophages, mature dendritic cells, and natural killer cells after damage, infection, or other kinds of triggers. It acts as a main character in PV by interfering with homeostasis, by compromising the normal immunosuppressive phenotype and enhancing inflammation. The link between HMGB1 and its receptors stimulates RAGE expression, inducing a Th17 shift in subjects affected by psoriasis and, in turn, sustaining the inflammatory condition (Figure 1). Borsky et al. found an interesting correlation between HMGB1 and psoriasin. This relationship was judged as a signal of their simultaneous release during the psoriatic autoimmune process. Moreover, they could be released after the death of specific cells [5]. HMGB1 released from skin, in particular from keratinocytes, may endorse the recruitment of inflammatory factors on site such as IL-18, replicating an autocrine loop in keratinocytes. The loop described could promote the onset of psoriasis [17]. Further results endorsed the endocrine alarmin pro-inflammatory hypothesis. A clue was the link between PV and metabolic syndrome and HMGB1 high levels in obese patients [26]. For this reason, some researchers proposed that HMGB1 could act as an adipocytokine, mimicking an innate proinflammatory molecule in patients with a high BMI. These findings were supported by HMGB1 correlation with other inflammatory markers of metabolic syndrome [27]. Therefore, the hypothesis that psoriasis is worsened by high levels of the alarmin is supported by the data reported above, suggesting an interesting therapeutic application.

### 4.1. Clinical Application as a Therapeutic Target

Borsky et al. focused on other interesting alarmins to support HMGB1 results. They found significantly increased levels of IL-33 in PV patients and reported a consistent link between HMGB1 and IL-33 in healthy controls [5]. S100A7 was increased and they observed a relationship with HMGB1. The same result was obtained for S100A12. According to their results, targeting these molecules is of importance to contain inflammation. For example, anti-IL-33 monoclonal antibodies have provided promising results in other skin conditions linked to inflammation, such as atopic dermatitis (AD). A phase 2a trial with etokimab, a humanized IgG1/ kappa anti-IL-33 monoclonal antibody, was performed with 12 moderate-to-severe adult AD patients. Etokimab was given at an SD of 300 mg intravenously. Twelve patients were observed for 140 days. All 12 patients achieved at least EASI-50 after an SD of etokimab, and three patients achieved IGA 0/1. Data reported endorse etokimab as an effective biologic in AD, although it is at an initial investigational stage [28]. Monoclonal antibodies demonstrated their potential on several other inflammatory conditions [14,29]. For this reason, the HMGB1 blockade may represent a new direction in the suppression of psoriasis [12]. Blocking the proinflammatory function of the HMGB1–IL-18 axis may be useful for psoriasis treatment in the future [17]. More data may encourage researchers to evaluate a trial in animals and, in the near future, in patients affected by psoriasis.

### 4.2. Role of Oxidative Stress

Alarmins, and in particular HMGB1, are released after cellular damage [9]. For this reason they have a major role in the chronic inflammatory loop in several diseases [30]. In this scenario, oxidative stress is intimately linked to cell damage and immune recruitment. Psoriasis is no exception [2]. The epidermis is a particularly sensitive tissue to free radicals. Once exposed to ROS, there is an accumulation of oxidation products which recruit immune system cells such as monocytes, macrophages, and neutrophils. The pro-inflammatory mediators released cause damage and stimulate production of other ROS, which lead to molecular alterations and to a re-amplification of the inflammatory response [2]. In this detrimental loop, alarmins act as catalysts, released after cell destruction. Controlling HMGB1, together with following an antioxidant healthy lifestyle, including a diet rich in antioxidants and nutraceutical intake, could greatly ameliorate PASI score and patient life quality [2,31].

## 5. Conclusions

PV patients had significantly increased levels of HMGB1 in lesional skin and serum. In some cases, this was linked to other pro-inflammatory markers and alarmins such as IL-33, S100A7, and S100A12. In most cases these parameters were correlated to PASI score. The discovery of the relationship between HMGB1, psoriasis and chronic inflammation is arousing much interest. First, alarmins could be useful as diagnostic biomarkers and monitoring substances for treatments. Second, initial data have demonstrated that blocking HMGB1 is effective in ameliorating psoriasis similar to blocking some other alarmins (e.g., IL-33) in other chronic inflammatory skin diseases (e.g., atopic dermatitis). Focusing on this approach could be valuable in terms of therapeutic options for counteracting immune related diseases in a way unthinkable until few years ago.

## Figures and Tables

**Table 1:** HMGB1 serum and tissue concentration and effects in the different types of psoriasis.

Alarmin	Author/Year	Tissue	Sample Size	Disease	Effects	Signaling
HMGB1	Chen 2017 [12]	Skin/blood	Mice model	Imiquimod-induced psoriasis	Worsening of psoriatic lesions after the injection of HMGB1	Enhanced presence of CD3+ T cells, myeloperoxidase + neu-trophils and CD11c+ dendritic cells; higher number of cd T cells, and augmented mRNA levels of IL-6, TNF-a, IFN-c and IL-17
HMGB-1	Watanabe 2020 [13]	Skin/blood	10 patients	Generalized pustular psoriasis (GPP)	Elevated serum HMGB-1 levels; affected skin had a high HMGB-1 expression in GPP and PV	Correlation between blood HMGB-1 levels and the Japanese severity score. HMGB-1 high levels in the cytoplasm
HMGB-1	Watanabe 2020 [13]	Skin/blood	10 patients	Psoriasis vulgaris	Serum HMGB-1 levels increased significantly; Affected skin had a high HMGB-1 expression in GPP and PVHMGB-1 high levels in the cytoplasm	HMGB-1 high levels in the cytoplasm
HMGB1	Borsky 2020 [5]	Blood	63 patients	PV	HMGB1 significantly elevated. No significant correlation with PASI.	Significant correlation between HMGB1 and psoriasin
rHMGB1 (recombinant HMGB1)	Wang 2020 [14]	Intradermal	mice	psoriasis-associated inflammation	Epidermal thickening and substantial cell infiltration	Induced Cxcl1, Cxcl2, Ccl20, S100a7, S100a8, S100a9, and Defb2 (defensin beta 2) associated with innate immunity (Il1b and Tnf), cytokines in the IL23A-IL17A axis (Il23a, IL17a, and IL22), transcription factors (Rorc/Rorγt) and the neutrophil membrane marker Ly6g
HMGB1	Kamel 2017 [15]	Blood	50 patients	Psoriasis	Significantly higher HMGB1 levels in patients. Positive correlation between PASI score and serum level of HMGB1	HMGB1 acts like an adipocytokine, an innate proinflammatory mediator. Higher HMGB1 levels among psoriatic patients with metabolic syndrome.
HMGB1	Wang 2017 [16]	Skin	12 patients	PV	HMGB1 expression in epithelial intercellular spaces increased. HMGB1 expression in psoriasis significantly increased.	HMGB1 diffuse expression in the nuclei, with low expression in the cytoplasm of the squamous epithelium. Extracellular HMGB1 occasionallypresent in the epithelium intercellular spaces. In the nuclei and cytoplasm of associated inflammatory cells and vascular endothelial cells, moderate HMGB1 expression.
rHMGB1	Zhang 2016 [17]	Keratinocytes	-	PV	11 inflammatory factors (IL18, IL1A, IL6, IL7, IL24, IL5, CXCL1, CX3CL1, TNFSF11, VEGFA) were shown to be upregulated by HMGB1 in keratinocytes,	Interleukin (IL)-18 showed the greatest change. The activation of the NfkB pathway and inflammasomes accounted for HMGB1-induced IL-18 production and release.
HMGB1	Zhang 2016 [17]	Skin	Mice	Imiquimod-induced psoriasis	HMGB1 and downstream IL-18 contributed to psoriasiform dermatitis	-
HMGB1	Bergmann 2016 [18]	Blood	90 psoriatic patients	PV	HMGB1 levels are significantly increased with disease progression	Increased level of HMGB1
HMGB1	Strohbuecker 2019 [19]	Skin	22 patients	PV	Increased staining for HMGB1 in the dermis of psoriatic plaques	Histocompatibility complex class III-encoded DNA and HMGB1 RAGE, induced by HMGB1, were highly expressed on psoriatic CD8+ T cells and CD4+ Treg. High RAGE levels, on the cell surface of keratino- cytes.
HMGB1	Chen 2013 [20]	Blood/skin	51 patients	PV	HMGB1 significantly higher; it correlated with PV severity and PASI score.	HMGB1 in normal skin was mostly limited to the nucleus. High cytoplasmic expression of HMGB1 in the epidermis in lesional skin of PV patients.

**Table 2:** Effects of HMGB1 neutralizing by experimental approaches or conventional therapies in psoriasis.

Alarmin	Author/Year	Tissue	Sample Size	Disease	Effects	Signaling
Anti-HMGB1 (anti-HMGB1 monoclonal antibody or HMGB1 inhibitor glycyrrhizin)	Chen 2017 [12]	Skin/blood	Mice model	Imiquimod-induced psoriasis	Mild clinical and tissue evolvement	It reduced the number of cd T cells, suppressed the mRNA expression of IL-6, TNF-a, IFN-c and IL-17
TNF-a inhibitors and one with granulocytapheresis (against HMGB1)	Watanabe 2020 [13]	Skin/blood	7 patients	GPP	Clinical improvement (GPP severity score, 0 or 1) after systemic treatment.	HMGB-1 significantly reduced after systemic treatment. Positive correlation between HMGB-1 and the GPP severity score
Injection of lentivirus expressing a small hairpin RNA (shRNA) against Hmgb1 (Hmgb1 shRNA)	Wang 2020 [14]	Subcutis	mice	Imiquimod-induced psoriasis	Epidermal thickening and inflammation	Deregulation of psoriasis-related genes
HMGB1-neutralizing antibodies	Zhang 2016 [17]	Peritoneally injected	Mice	Imiquimod-induced psoriasis	Thinner epidermis than controls	T-helper 17 immune response inhibited by both HMGB1 and IL-18 blockade
TNFα-inhibitors, fumaric acid esters and methotrexate	Bergmann 2016 [18]	Blood	90 psoriatic patients	PV	HMGB1 was reduced during therapy	TGF-β1 and IL-23 were decreased

## Data Availability

Not applicable.
